# The Role of microRNA in Pancreatic Cancer

**DOI:** 10.3390/biomedicines9101322

**Published:** 2021-09-26

**Authors:** Beata Smolarz, Adam Durczyński, Hanna Romanowicz, Piotr Hogendorf

**Affiliations:** 1 Laboratory of Cancer Genetics, Department of Pathology, Polish Mother’s Memorial Hospital Research Institute, 93-338 Lodz, Poland; hanna-romanowicz@wp.pl; 2 Department of General and Transplant Surgery, N. Barlicki Memorial Clinical Hospital, Medical University of Lodz, 90-153 Lodz, Poland; adam.durczynski@umed.lodz.pl (A.D.); piotr.hogendorf@umed.lodz.pl (P.H.)

**Keywords:** miRNA, pancreatic cancer, therapeutic potential

## Abstract

MicroRNAs (miRNAs) are small ribonucleic acid molecules that play a key role in regulating gene expression. The increasing number of studies undertaken on the functioning of microRNAs in the tumor formation clearly indicates their important potential in oncological therapy. Pancreatic cancer is one of the deadliest cancers. The expression of miRNAs released into the bloodstream appears to be a good indicator of progression and evaluation of the aggressiveness of pancreatic cancer, as indicated by studies. The work reviewed the latest literature on the importance of miRNAs for pancreatic cancer development.

## 1. microRNA–Structure and Function

Cancer is a significant civilization problem of our age. Scientists continue to search for new factors responsible for the process of carcinogenesis. In 1993, Victor Ambros, Rosalind Lee, and Rhonda Feinbaum discovered that one of the *Caenorhabditis elegans* genes, lin-4, involved in controlling the larval development of this non-parasitic nematode, did not encode protein, but a pair of short RNA—about 22 and about 61 base pairs. The RNA in question, in turn, was antisense complementary to many places at the end of the 3′UTR lin-14 gene [1]. Further studies have shown that lin-4 gene products regulated the lin-14 gene by reducing the amount of LIN14 protein, while maintaining the mRNA concentration of lin-14 [2]. Finally, it was argued that these short RNA had an inhibitory effect on the action of lin-14, thereby regulating the onset of the transformation from the first larval stage of *C. elegans* to stage two [2]. RNA with a length of about 22 base pairs was considered the first of a rich family of microRNAs, mainly performing regulatory functions [2]. The following years brought the discovery of new microRNA molecules. Their presence has been observed in numerous organisms, not only mammals, but also insects, nodules, or plants [1]. The vast majority of microRNAs remain evolutionarily conservative [1,2]. Individual microRNAs are also typically found in specific cells, e.g., miR-122 in liver cells [1].

Genes for microRNAs are located in the genome in a very diverse way. They are part of operons, occur between or are part of protein coding sequences [2]. They happen in exons, introns or sequences that are not translated [2]. They may constitute an independent transcription unit [2]. As a component of the intron, they can be transcribed together with the entire gene that encodes the protein, resulting in microRNAs and mRNA (pre-mRNA) [1]. Genes for microRNAs are transcribed by polymerase II or III RNA [1,2].

Genes for microRNAs are often organized in clusters that are transcribed as polycistron transcription units [3]. They can occur between protein coding sequences and function as stand-alone transcription units, they can also be located in coding sequences [4].

This arrangement of the transcription unit can lead to the simultaneous formation of miRNA and mRNA transcripts [5]. MiRNA genes are organized in a manner characteristic of polymerase II and III, which transcribes small RNA genes [6,7]. The formation of miRNAs consists of several stages. The first is transcription, which leads to the formation of the primary pri-miRNA transcript. Another is the treatment of pri-miRNA, as a result of which pre-miRNA is formed. Both stages occur in the cell nucleus. Then the pre-miRNA is transferred to the cytoplasm, where it is subjected to processes leading to the formation of a mature, functional miRNA molecule with a length of ~20 nt [8].

The primary transcripts (pri-miRNAs) form interconnected hairpin structures, polyadenylate and with a cap at the end of 5′. They contain multiple parent loops. Pri-miRNAs can be several kilos long base pairs [9]. The double-stranded structures of pri-miRNAs are recognized by the nuclear protein DGCR8 (in Pasha Invertebrates). This protein is bound to Drosh ribonuclease, an enzyme belonging to the RNaz III group. Together they form a microprocessor complex that is involved in the processing of primary miRNA transcripts in the cell nucleus. In this complex, DGCR8 (Di George syndrome critical region gene 8) attaches to the single-stranded pri-miRNA ends and orients the catalytic domain of the ribonuclease in such a way that it can cut through the transcripts and release the structures of pre-miRNA hairpins with a length of ~60–100 nucleotides [10]. In this form, they are transferred to the cytoplasm by Exportine 5 (Exp-5). These are transport proteins that need energy to function properly. It is obtained from GTP bound to the RAN protein. Exportins 5 belong to the group of caryopherines that require dinucleotide overhangs at the 3′ end, formed after the treatment of pri-miRNA in the cell nucleus by the Drosha enzyme [11]. If the genes for miRNAs are located in introns (myrtrons), then the formation of pre-miRNAs from primary transcripts occurs bypassing the microprocessor and is associated with cutting out introns from mRNA [12]. In the cytoplasm, the treatment of pre-miRNAs by the Dicer enzyme leads to the formation of a double-stranded molecule with a length of ~20 nucleotides. Dicer belongs to the RNaz-III group; like exportins, it only functions properly for dsRNAs containing dinucleotide overhangs at the 3′ end. As a result of the activity of the Dicer enzyme, double-stranded miRNA-miRNA* duplexes are formed in the cytoplasm. One thread in the duplex is called the leading strand, the other the passenger strand and is marked with an asterisk. Both strands are connected to each other for some time, with some nucleotides in the duplex being able to remain unpaired, which is related to the possibility of differences in the ribonucleotide sequence between these strands. Until recently, it was thought that the passenger strand is degraded in most cases, but studies have shown that both strands can be functional [13,14]. The active form of miRNA to ssRNA, built into the protein miRNP complex is called RISC (microRNA induced silencing complex). It consists of many proteins, but the main role in miRISC is played by Ago (Argonaute) proteins. Owing to them, it is possible to degrade the target mRNA or repression of translation, or a combination of both processes. The Argonaute family of human proteins includes eight genera. Human Ago2 has been shown to have endonuclease activity [15]. Ago proteins are the basic component of silencing complexes, acting transcriptively or post-transcriptionally. Recently, Ago2 has been shown to build silencing complexes with pre-miRNA molecules. Such in vitro active combinations are a custom RISC model [16].

The mechanism of action of miRNA molecules is related to the post-transcriptional regulation of gene expression, which is possible due to the complementarity of base pairs with informational RNA molecules [17]. Gene silencing can be done either by degrading a specific mRNA or by inhibiting the translation of the transcript. MiRNA molecules are attached to the 3′ non-translational region (3′UTR) of the target mRNA [1]. If there is complete complementarity between the miRNA molecule and a specific mRNA sequence, the Ago2 protein can split the mRNA molecule leading to its direct degradation. In the case of incomplete complementarity, muting is carried out on the principle of blocking translation [18]. Unlike plants, in animals, the mechanism associated with the degradation of informational RNA occurs much less frequently, and gene silencing as a result of translation inhibition dominates. In mammals, it is estimated that less than 5% of the target sequences for miRNAs are split by binding these regulatory molecules [19]. When the miRNA degrades the target transcript, the observed cutting sites are identical to the degradation catalyzed by small interfering RNA (siRNA)–fission usually occurs between nucleotides at positions 10 and 11 of the resulting siRNA:mRNA duplex [20,21,22]. The dissement sites do not change even if the miRNA is not perfectly paired with the target sequence of the mRNA at its 5′ end. After degradation of a specific transcript, miRNA can recognize and catalyze the cleavage of subsequent molecules of the matrix RNA, since during this process it is not damaged [23]. The detailed course of the translation blocking mechanism is not exactly understood. It is assumed that the target sequences for miRNAs can be complexed on polysomes or attracted to cellular P corpuscles, where they are removed from the translational complex and possibly destroyed [24]. The choice of the appropriate mechanism does not depend on whether a small silencing RNA molecule is formed as siRNA or miRNA, but is determined by the type of target sequence. Once attached to the cytoplasmic RISC complex, the miRNA will uniquely lead to transcript cleavage, in the case of sufficient mRNA complementarity.

Inhibition of translation will occur in a situation of incomplete matching of mRNA and miRNA, but with the appropriate arrangement and distribution of complementary sites [20,21]. Disturbances in the expression of microRNAs are observed in various types of tumors. Almost 50% of miRNA genes are located in fragile sites. Mutations in these areas are often linked to cancer. This indicates a significant function of microRNAs in the formation and progression of tumors [25]. Brittle places are areas where chromosome fragments are lost or rearranged with high frequency. Such changes are often observed in cancer cells. The expression of microRNA genes located near such regions may be disturbed. An example is the miR-15a and miR-16-1 genes located on the long arm of chromosome 13 in the 14.2 area, where deletion often occurs. Reduced levels or complete absence of miR-15a and miR-16-1 are found in many patients with chronic B-cell lymphocytic leukemia, prostate cancer, mantle cell lymphoma, and multiple myeloma [26]. Changes in the expression level of about 200 miRNAs are observed in many types of cancer [27].

Studies show that miRNAs affect the course of processes of fundamental importance for the proper functioning of the body. These processes include cell division, proliferation, differentiation, cell apoptosis, as well as blood vessel formation, or, finally, neoplasm [28]. The altered expression of individual miRNAs has been demonstrated in a number of cancers, which may indicate the oncogenic or suppressive potential of the molecules [28,29].

As research indicates, the repression of miRNAs released into the bloodstream appears to be a good indicator of progression and evaluation of the aggressiveness of pancreatic cancer. A number of different microRNA molecules show abnormal expression in the course of cancer (Table 1). In one experiment, a study of patients’ plasma showed as many as 37 types of reduced expression miRNAs and 54 miRNAs over-expressed [30].

The relationship between the expression of miRNA family and the cell cycle, including the disturbed cycle of the cancer cell, certainly requires further analysis. There is no doubt, however, that the molecules in question have great prognostic, predictive, and therapeutic potential.

Neoplastic diseases, despite the existence of prognostic therapies, are still one of the most dangerous diseases. According to the 2018 WHO report, they are the second leading cause of death. Among the neoplasms causing the highest mortality in humans, there are mainly lung, liver, breast, and digestive system neoplasm’s [31].

The idea of involvement of microRNAs in the process of tumorigenesis has been positively verified. It has been proven that the expression of more than 30% of genes in human beings is controlled by miRNAs. It is also known that one of the factors that contribute to the cancer is changes in the level of expression of miRNA in cancer-altered tissue as well as in surrounding tissues.

Neoplastic cells, regardless of their tissue origin, are characterized by a low degree of differentiation, increased proliferation, faster growth and changes in systems responsible for cell death. Research shows that miRNAs are able to control these processes [32].

Many miRNA genes are located in sensitive (especially vulnerable) sites of the genome, which are frequently amplified or deleted in the process of carcinogenesis [33]. Impaired miRNA expression can cause the development of many tumors (Table 2) [34,35,36,37,38,39,40,41,42,43,44,45,46,47,48,49,50,51,52,53,54,55,56,57,58,59,60,61,62,63,64].

miRNAs have recently been discovered, but research into their properties and possible applications is still ongoing and has huge prospects. One aspect of their use is the field of cancer, including pancreatic cancer. Disturbances in their properties or structure due to significant functions performed in the body can contribute to carcinogenesis. Variable levels of miRNA expression can be detectable in the blood of patients. Many studies confirm that certain miRNAs are not only related to the type of cancer, but also its developmental stage.

To sum up, these findings may result in the design of an effective, non-invasive molecular biomarker for cancer detection, prediction, and monitoring effects of treatment. Further research into their specificity, in turn, may make it possible to produce effective drugs to fight developing cancers.

## 2. The Role of microRNAs in Pancreatic Cancer

In Europe, pancreatic cancer (PCa) is the seventh most common cancer. In the European Union, pancreatic cancer is diagnosed in 11.6 men per 100,000; this frequency ranges from 4.7 (Cyprus) to 17.2 (Hungary), causing pancreatic cancer to cause the deaths of approximately 35,000 men per year. In women it affects 8.1 per 100,000, from 2.1 (Cyprus) to 11.4 (Finland). It is also the cause of death for 35,000 women a year [31]. The frequency of diagnosis of new cases increases with age, with in most cases the diagnosis occurs in patients over the age of 65 years. Since the disease often develops unnoticed for a long time, the diagnosis often occurs at the time of its spread to other organs. This is the fifth most common cause of cancer death.

The main causes of pancreatic cancer are gene mutations (KRAS, p53, p16 CDKN2, DPC4/Smad4, BRCA2), smoking, age, obesity, chronic pancreatitis. About 25% of pancreatic cancer patients are or have been long-term smokers [31]. This addiction has a stronger effect if you have one of the genetic teams listed above. The risk of developing pancreatic cancer increases with age. Pancreatic cancer is most commonly diagnosed between the age of 60 and 80. The risk of pancreatic cancer may increase slightly with an increase in body mass index (BMI) has been proven. Chronic pancreatitis, lasting several decades, increases the risk of pancreatic adenocarcinoma. This risk is increased by smoking and genetic factors.

The components of the diet shape the composition of the microbiota inhabiting the digestive tract, which play an important role in maintaining human health. There is strong evidence that diet, microbiota, and its metabolites significantly affect the epigenome, particularly by modulating microRNAs. This group of small non-coding RNAs maintains cellular homeostasis; however, any changes leading to impaired miRNA expression contribute to the development of various pathologies, including cancer. The imbalance of the intestinal microbiota caused by diet is associated with the development of tumors. Cancer-related deaths can be attributed to dietary factors [65].

Hence, dietary patterns appear to be potentially effective in inducing or preventing cancer directly or indirectly by modulating gut microbial composition and microbial metabolism. Bacteria and their metabolites can affect various signaling pathways, cause splitting of double-stranded DNA, promote apoptosis, alter cell differentiation, induce inflammation, and help maintain the body’s homeostasis. The microbiota includes all living microorganisms, including mainly bacteria, fungi, and viruses forming a microbiome [66].

The interaction of miRNA regulation and microflora has only recently been appreciated. Evidence indicates that human miRNAs from feces, such as miR-515-5p and miR-1226-5p, target specifically the bacterial genes that control bacterial growth and thus directly shape the overall composition of the gut microbiota. Studies show that gut microbes can control host miRNA expression and influence cancer progression. For example, butyrate produced by the gut microbiota induces the expression of miRNAs such as miR-22 and reduces others, including miR-106b and miR-92a. Therefore, non-coding RNA mechanisms involving inter-specific gene regulation are extremely important for maintaining homeostasis and human health [66].

Epidemiological observations can provide insight into diet, lifestyle, and genetic factors that influence miRNA expression. Such observations about nutritional factors are only beginning to be studied. One example relates to the association of miRNA expression with various exposures, including alcohol consumption, and clinical features associated with squamous cell carcinoma of the head and neck [67]. In tumor tissue from 169 cases, miR-375 expression was shown to increase significantly with alcohol consumption and show higher expression in tumors of pharyngeal and laryngeal origin compared to oral tumors. Research into the relationship between dietary variables and circulating miRNA in people at high risk of certain cancers is ongoing [67].

Dietary folate has been found to modulate miRNA expression in various model systems, which may be related to folate activity in cancer prevention and risk. Human lymphoblastoid cells grown in folic acid-deficient medium induced significant changes in levels of 24 miRNAs, including hsa-miR-222 [68]. When folate was re-added to the medium, the miRNA expression profiles returned to the control cell profiles. These results suggest that dietary modulation of miRNA expression is reversible. In addition, miR-222 expression increased in vivo in human peripheral blood from people with low folic acid status compared with those with the highest folic acid status. These data suggest that abnormal miRNA expression may be potential biomarkers of nutritional status in humans, as well as participants in cancer prevention [68].

Studies prove that diet is crucial in the development of cancer. The consumption of fresh fruits, vegetables, nuts, and beverages (i.e., tea, wine), which are rich in polyphenols, modulates the expression of miRNA and, in some cases, epigenome, having a protective effect [69].

Polyphenols are the main class of metabolites that possess antioxidant properties, free radical sweeping properties, and a noteworthy cancer protection effect. More than 500 polyphenols have been identified, including classes of phenolic acids, flavonoids, schistbenes, tannins, lignans, quinones, and curcuminoids. Many of these compounds directly regulate miRNAs, which are closely related to human cancers. In particular, dietary polyphenols have been intensively studied: resveratrol, elagitani and ellagic acid, epigallocatechin 3-gallate, genistein, curcumin, and diindolylmethane [69].

They exert their action on inhibiting cancer and protecting against cancer by regulating miRNA expression. These edible compounds are promising therapeutic drugs and disease prevention agents to administer to the general population, since foods rich in these anti-cancer polyphenols are readily available, non-toxic, and affordable.

Resveratrol belongs to the class of stilbenoid polyphenolic compounds commonly found in grapes, wine, peanuts, cocoa, berries, and cranberries. This polyphenol has an antioxidant and protective effect against various malignant tumors, including pancreatic cancer. Resveratrol has been shown to inhibit the growth of pancreatic and bladder cancer cells and induce apoptosis by suppressing pro-oncogenic expression of miR-21 and anti-apoptotic factor BCL2.

3,3′-Diindolylmethane (DIM) has been shown to have anti-cancer properties in various tissues, in part by regulating cancer-related miRNA expression. Studies have shown a significant reduction in the levels of suppressor miRNAs miR-200b, miR-200c, let-7b, let-7c, let-7d, and let-7e in pancreatic cancer cells resistant to the drug gemcitabine. DIM treatment significantly increased the expression of miR-200 (miR-200a, miR-200b, miR-200c), let-7 family, and miR-146a, improving the effects of gemcitabine treatment [69]. DIM treatment of pancreatic cancer cells has been shown to result in a decrease in miR-221 expression.

The growth and migration of pancreatic cells after treatment was attributed to an increase in the expression of target miR-221 proteins such as PTEN, cyclin-dependent kinases (CDK) p27kip1 and p57kip2, as well as the upwardly regulated p53 apoptosis modulator (PUMA). Interestingly, a synthetic curcumin analogue was shown to give similar results by miR-221 regulation [69].

Curcumin (diferuloylmethane), a component of the Spice Curcuma longa (or turmeric), has been found to modulate cancer signaling pathways, possibly through miRNA expression. Curcumin has been found to increase levels of 11 and reduce levels of 18 miRNAs after 72 h of incubation in human pancreatic cancer cells [70].

In another study, curcumin increased the expression of miR-15a and miR-16 in MCF-7 cells [71]. Both miR-15a and miR-16 inhibited Bcl2 expression, thereby inducing apoptosis. This proapoptotic activity was confirmed by silencing miR-15a and miR-16 with anti-miR-15a and anti-miR-16 oligonucleotides, which restored Bcl2 expression. Curcumin has also been shown to promote apoptosis in human lung adenocarcinoma cells by signaling miR-186*. Curcumin has been shown to inhibit the binding of activating protein-1 (AP-1) above pri-miR-21, which reduces miR-21 expression and induces expression of the tumor suppressor Pdcd4, the target of miR-21 [72]. In addition, curcumin inhibited tumor growth, invasion, and metastasis by inhibiting miR-21 transcription regulation. Thus, curcumin appears to modulate miRNAs that target proliferation, apoptosis, invasion, and metastasis depending on the cellular context.

MiRNAs have been shown to be epigenetically regulated. Studies cited in the Anteneh et al. review paper confirm the concept of epigenetic modifications targeting miRNAs [66].

Epigenetic changes can be used in the diagnosis and treatment of pancreatic cancer. However, research on the effect of epigenetic miRNA modulation in pancreatic cancer is in small numbers.

Overextension of several miRNAs that are generally suppressed, miR-29a, miR-29b, miR-103, miR-107, and miR-320, has been shown in pancreatic cancer cell lines as a result of treatment with a histone deacetylase inhibitor, trichostatin A, or a decitabin hypomethylating agent. Forced expression of miR-107 in MiaPACA-2 and PANC-1 cells inhibited their growth in vitro and suppressed the alleged target of miR-107, cyclin-dependent kinase 6, thus providing a functional basis for epigenetic inactivation of this miRNA in pancreatic cancer.

An examination of the epigenetic regulation of various miRNAs in a wider panel of tumors (including mammary tumors, colorectal tumors, pancreatic tumors, and soft tissue sarcomas) showed consistent methylation of CpG miR-34a and miR-34-b/c in tumor tissues.

Inducing miR34a overexpression by treatment with decitabin or vorinostat, a histone deacetylase inhibitor, resulted in cell cycle arrest, invasion, and inhibition of pancreatic cancer growth. It was found that methylation of CpG islands leads to miR-1247 hypo-expression. Unlike non-cancerous tissues, pancreatic cancer contains highly methylated miR-124-1, miR-124-2, and miR-124-3. Hypermethylation of these genes has been linked to poorer survival for pancreatic cancer patients.

Overexpression of MRG domain binding protein (MRGBP) has been documented in malignant tumors, including pancreatic cancer. The expression of MRGBP is regulated downwards by miR-137, its loss leads to overexpression of MRGBP. Adverse tumor features have been observed in patients with pancreatic cancer who experienced a loss of miR-137 [66].

Re-expression of miR-137 significantly blocked the migration and invasion of pancreatic cancer cells. Therefore, the loss of miR-137 may act as a new tumor promoter, facilitating overexpression of MRGBP in pancreatic cancer. Higher serum levels of miR-21 as a result of promoter histone acetylation are observed in patients with gemcitabine-resistant pancreatic cancer compared to patients with gemcitabine-sensitive tumors. In cancer cells after treatment with gemcitabine, miR-21 levels increased significantly, causing a marked increase in invasiveness and metastatic potential by activating Akt and reducing the homologue of phosphatase and tensin (PTEN). These changes were reversible after transfection of anti-miR-21 [66].

MiR-663b expression is epigenetically suppressed (on H3K4me3 and H3K27me3 promoters) by the long non-coding RNA, antisense RNA homeobox transcript (HOX) (HOTAIR). Overexpression of miR-663b resulted in inhibition of cell proliferation, invasion and migration, and induced apoptosis. Re-expression of miR-663b or HOTAIR silencing blocked tumor growth by targeting insulin-like growth factor 2 in pancreatic cancer.

MiRNA deregulation supports the microenviral environment of pancreatic cancer. The desmoplastic reaction, characterized by a dense concentration of extracellular matrix proteins, activated pancreatic stellate cells and immune cells surrounding the tumor, is a well-described feature of pancreatic adenocarcinoma. This has been identified as a barrier to the delivery of anticancer drugs, as one explanation for poor treatment outcomes. In experiments in which human pancreatic stellate cells were cultured together with pancreatic adenocarcinoma cells, increased expression of fibrosis-related genes was observed to reduce the expression of let-7d miRNA [66].

Inhibition of let-7d miRNA resulted in increased expression of genes associated with fibrosis (smooth muscle α-actin, β-growth factor platelet-derived receptor, and collagen type I alpha 1 chain (COL1A1)). Literature data showed that let-7d expression was consistently reduced in pancreatic cancer compared to normal tissue [66]. In addition, patients with pancreatic ductal adenocarcinoma have been shown to have lower serum let-7d levels compared to healthy controls. Decreased let-7d expression correlated with poor overall survival in patients treated with gemcitabin-based chemotherapy [66].

The accumulated evidence confirms that miRNAs may play a key role in the pathogenesis of pancreatic cancer, influencing important genetic changes such as KRAS, Tp53, and TGFβ/SMAD, and supporting the unfavorable tumor microenvironment.

miRNAs represent an attractive therapeutic target and important biomarkers that can be used in diagnosis, prediction of response to treatment, and prognosis of pancreatic ductal adenocarcinoma. Their ubiquity and incomplete understanding of their effects on normal cellular mechanisms and intercellular interactions limit their current clinical usefulness and require further research. Despite extensive preclinical work, there is still a lack of practical clinical application.

## 3. microRNA–Iagnostics Potential

Pancreatic cancer is considered to be the most malignant tumor with a poor prognosis, due to the rapid course and absence of symptoms at the initial stage of the disease [34]. Its late detection, lack of effective tools for monitoring its development, early and aggressive local invasion, and high metastasis potential make this cancer with one of the worst rates of overall survival [73]. That is why such an important problem is the discovery and introduction into general diagnostic practice of effective and easily detectable biomarkers that would allow determining the presence of the disease at its initial stage and thus significantly improving the survival rates of patients.

Among the molecules with significantly increased levels of expression compared to control samples, attention is most often drawn to oncogenic miR-16, miR-21, miR-155, miR-181a, miR-181b, miR-196a, and miR-210 [74,75].

In contrast, the let-7 molecules are under-expressed [75]. Wang et al. suggested that the expression study of four plasma miRNAs (miR-21, miR-155, miR-196a, miR-210) could be an effective tool for distinguishing pancreatic adenocarcinoma patients from healthy subjects with high sensitivity and specificity [75]. miR-155 has also been shown to be a good biomarker for early pancreatic cell neoplasia and its oncogenic function has been associated with inhibition of the proapoptotic action of the tumor protein 53-induced nuclear protein 1 (TP53INP1), which increases the malignancy of cancer cells in vivo [76].

A great deal of attention is paid to the function of microRNA-21 and miR-221 in research into the development of pancreatic cancers. They function as oncogenes, which is associated with their over-expression in many different cancers. The target genes for miR-21 are i. a. phosphatase and tensin homolog (PTEN) and programmed cell death protein 4 (PDCD4), which have a suppressive effect, the reduced activity of which results in inhibition of tumor cell apoptosis and the acquisition of invasive properties. In addition, the expression of these two microRNAs has been shown to change with long-term conventional anticancer therapy. Therefore, drug resistance of pancreatic cancer may result from miR-21 over-expression and inhibition of the activity of genes regulated by this molecule in refracting cell lines [75].

One of the important factors helping to detect pancreatic cancer is the marker CA19-9. Attempts are currently being made to determine the diagnostic efficacy of this indicator in conjunction with other molecules in the serum that are potentially useful in diagnosing cancer.

Liu et al. measured Ca19-9 expression levels and selected microRNAs in cancer, chronic inflammation, and healthy subjects. They showed that a combination of biomarkers such as CA19-9, miR-16, and miR-196a is a very effective and non-invasive tool to identify cancer sufferers, especially at an early stage [75].

miR-210 may also have significant diagnostic potential for pancreatic cancer. The expression of this molecule is induced by oxygen deficiency at the tissue level and correlates with the inauspicious results of treatment in some cancers. Pancreatic adenocarcinoma has been shown to have increased levels of hypoxia, which in turn results in significantly increased levels of circulating miR-210, poorer prediction, and greater cell resistance to chemo- and radiotherapy [77].

Serum for pancreatic cancer patients has also been shown to over-express miR-200a and miR-200b molecules [78].

This is probably related to hypomethylation of genes that encode them. The molecular target of miR-210 is i.e., Smad-interacting protein (SIP1) gene that is muted in pancreatic cancer cells.

Its protein product inhibits the activity of kadherin E and is involved in epithelial-mesenchymal transformation. It was therefore hypothesized that the hypermethylation of the SIP1 gene promoter observed in patients may be related to abnormal expression of miR-210, despite the absence of clear evidence of the contribution of microRNAs to epigenetic processes [78]. Table 3 shows the expression of circulating microRNAs in pancreatic cancer.

Pancreatic cancer is one of the worst-looking solid tumors, due to usually the late diagnosis and lack of effective therapy. Identification of markers characteristic of individual cancer phenotypes is strategic for early diagnosis and the use of effective therapeutic methods.

In pancreatic cancer cells, increased gene expression for mucin 4 (MUC4—cell surface associated, mucin 4) is observed. This is associated with increased mobility of cancer cells and their ability to tissues infiltration and metastasis [79]. This is probably related to reduced levels of miR-150 expression in pancreatic cancer cells, as an inverse correlation was observed between the amount of MUC4 protein and the level of microRNA-150 expression. Computer analysis has shown that MUC4 can be the target gene for the miR-150 molecule, which is believed to be a tumor suppressor [80].

microRNA-150 also affects the expression of human epidermal growth factor receptor 2 (HER2). Extortion of miR-150 expression in pancreatic cancer cells has been shown to lead to a decrease in the expression of HER2 receptors as well as the level of their phosphorylation. The transfection of miR-150 molecules into cancer cells has also been shown to inhibit their ability to form clones, migrate and intrude on tissues, while increasing intercellular adhesion [80].

The miRNA-21 molecule functions as an oncogene in pancreatic adenocarcinoma and is observed to be over-expressed in a significant number of patients, which is associated with the occurrence of lymph node metastases and poorer prognosis [81].

miRNA-21 may affect the formation and development of an aggressive type of ductal pancreatic adenocarcinoma. miR-21 has the potential to silencing genes such as programmed cell death factor 4 (PDCD4-neoplastic transformation inhibitor) or tissue metalloproteinase inhibitor 3 (TIMP3), which can make the course of the disease much more aggressive [40]. The abolition of PDCD4 expression as a result of miR-21 overexpression inhibits the stimulation of cancer suppressors such as TP53, cyclo-dependent kinases, or the urokinase plasminogen activator receptor (u-PAR). This can lead to a decrease in cycle control and cellular differentiation, inhibition of apoptosis, intense cell divisions, and consequently to metastasis and disease progression [82].

Recent work indicates that high miRNA-21 expression affects the prognosis of patients with pancreatic cancer [83]. The inhibition of TIMP3 expression by miR-21 may be one of the causes of tumor growth and the acquisition of metastasis capacity by cells. In order for cancer cells to escape from the tumor into the bloodstream or lymphatic vessels and reach other tissues, they need to overcome the base membranes and tissue-cell structures formed, among other things, from collagen. These processes are facilitated by extracellular metalloproteinase (MMP) activity. Silencing the expression of tissue inhibitors of metalloproteinase (TIMP) increases their activity, which facilitates the formation of distant metastases [84].

CA 19-9 is one of the serous markers, the evaluation of which is used in the diagnosis of pancreatic cancer. However, it is little specific by non-specific expression in benign and malignant tumors, which can produce false positive and false negative results [85]. The combination of two research strategies, i.e., identification of the expression profile of selected circulating miRNAs and CA 19-9 level studies may be more effective in the diagnosis of tumors in the initial stages of the disease. miR-16 and miR-196a have been selected from a number of microRNAs because they show diagnostic potential for over-expression in pancreatic cancer cells [83]. By assessing the expression of these two microRNAs, patients with pancreatic cancer and people with chronic pancreatitis can be differentiated. This is very important in view of the possibility of rapid diagnosis of the disease and the selection of the most appropriate treatment [86].

miR-34a and miR-143/145 show reduced levels of expression in most cases of pancreatic cancer [75]. Studies were conducted using mouse xenographers, which had induced subcutaneous tumors with the properties of pancreatic tumors. They were characterized by reduced expression of microRNA-34a and microRNA-143/145. The introduction of plasmid vectors with embedded microRNAs into their cells has led to the inhibition of the growth of PCa tumors, an increase in apoptosis levels, and a decrease in the proliferation capacity of cancer cells [75].

This was done both by the ability of miR-143/145 to mute V-Ki-ras2 Kirsten rat sarcoma viral oncogene homologue (KRAS2) transcripts and by the involvement of miR-34a in the processes associated with the functioning of the TP53 protein. Perhaps further research will improve this strategy and will be used in the future as an alternative to traditional therapies [75].

Recent studies have shown that TSPAN1 expression levels are elevated in pancreatic cancer and that reducing its expression reduces the proliferation of pancreatic cancer cells in vitro and in vivo [87]. TSPAN1 expression was correlated with poor overall survival of pancreatic cancer patients. In addition, it has been demonstrated that TSPAN1 is a new positive macro-autophagy/autophagy regulator with reduced LC3-II and SQSTM1/p62 expression, inhibition of GFP-LC3 point formation and autophagous vacuoles. It was also demonstrated that TSPAN1 promoted the maturation of autophagy by directly binding to LC3 by two conservative LIR motifs. Mutations in the LIR TSPAN1 motives resulted in a loss of ability to induce autophagy and promote the proliferation of pancreatic cancer. Two conservative TCF/LEF-binding elements present in the TSPAN1 gene promoter region were detected, which was further verified by luciferasis activity and CHIP tests. Moreover, TSPAN1 was elevated by FAM83A through the canonical signaling pathway WNT-CTNNB1. In addition, it has been demonstrated that both TSPAN1 and FAM83A are direct targets of MIR454 (microRNA 454). In addition, it reveals the role of MIR454-FAM83A-TSPAN1 in the proliferation of pancreatic cancer cells in vitro and in vivo. Research suggests that elements of the MIR454-FAM83A-TSPAN1 pathway may be valuable prognosis markers or therapeutic targets for the pancreas [87].

Activation of Notch was detected in pancreatic ductal adenocarcinoma (PDAC) [50]. However, its role in PDAC metastases remains unknown. In this study, a Notch-dependent feedback circuit was detected between pancreatic cancer cells and macrophages, which contribute to PDAC metastasis. In this circuit, the miR-124 regulated the signaling of Notch in cancer cells, directly aiming at the Ligand Notch Jagged 1. Auto-boost Notch’s signaling promoted the recruitment and activation of macrophages into the cancer-supporting phenotype M2 through IL8, CCL2, IL1α, and paracrine uPA. In turn, activated IL6 from macrophages activated the oncogenic transcription factor STAT3, which directly suppressed the miR-124 genes through the conservative STAT3 binding site in their promoters, thus promoting the transition and invasion of epithelial-mesenchymal cancer cells. The disruption of this circuit suppressed liver metastases in mouse models. Thus, the study suggests that manipulation of this Notch-dependent circuit has therapeutic potential in the treatment of PDAC metastases. This study provided potential therapeutic targets and solid preclinical evidence for PDAC treatment by interrupting the feedback between cancer cells and macrophages with targeted inhibitors [88].

Exosomes play an important role in the tumor microenvironment and mediate the interaction between pancreatic cancer cells (PC) and matrix components, including pancreatic stellate cells (PSC), to regulate the progression of pancreatic cancer [56]. Primary PSC was isolated from PC patients and demonstrated that exosomes derived from PSC can be internalized by PC cells to promote cell proliferation. In addition, miR-5703 was identified in exosomes as a driver of cell proliferation, and its inhibitor inhibited exosome function to promote cell proliferation. Knockdown CMTM4 promoted PC cell proliferation, while CMTM4 over-expression inhibited PC cell proliferation in both in vivo and in vitro. CMTM4 suppressed the PI3K/Akt pathway by downregulation of PAK4. The findings suggest that PSC exosomal miR-5703 may target CMTM4 in PC cells and promote cell proliferation due to the PI3K/Akt pathway activated by PAK4 [89].

Alpha 1 subunit of prolyl 4-hydroxylase (P4HA1) plays a key role in modulating the extracellular matrix component and promoting tumor progression by altering tumor adhesion, migration, and other biological behaviors in some cancers. However, the expression pattern, biological function, and mechanism underlying pancreatic cancer remain largely unclear. Hu et al. found that mRNA and P4HA1 protein expression were significantly higher in pancreatic cancer tissues than in normal tissues [90]. The high expression of P4HA1 correlated with bad clinical-pathological characteristics. LINC01503/miR-335-5p/P4HA1 may mediate the action of P4HA1 in promoting pancreatic cancer progression.

Pancreatic cancer (PC) is a severe disease with the highest mortality among various cancers. An effective and accurate way of predicting the survival of PC patients urgently needs to be found.

Gene set variability analysis (GSVA) was used to determine and validate the prognostic signature of the miRNA-based path for PC (miPPSPC) and the prognostic signature of the PC mRNA (mPPSPC) path in independent data sets [91]. MiPPSPPC has been optimized by combining clinical parameters. MiPPSPPC, optimized miPPSPC and MSPSPC have been developed and validated to predict the survival of PC patients and have shown excellent predictability. Four metabolic pathways and one oxidative stress pathway have been identified in miPPSPPC, while the IPPSPPC has identified linoleic acid metabolism and the pentosophosphate pathway. Key factors in the pentosophosphate pathway and linoleic acid metabolism, G6PD and CYP2C8/9/18/19 respectively, are associated with the survival of PC patients according to tissue microarray studies. In this way, miPPSPPC, optimized miPPSPPC and MSPPSPPC can effectively and accurately predict the survival of PC patients. Metabolic and oxidative stress pathways may participate in PC progression.

Long non-coding RNA (lncRNAs) are usually deregulated in pancreatic cancer progression. The Cancer Genome Atlas (TCGA) database was used to analyze the relationship between patient survival and LINC00261 or miR-23a-3p levels in pancreatic cancer. The low level of LINC00261 indicates a low probability of survival of patients with pancreatic cancer [92]. LINC00261 levels were reduced in pancreatic cancer cells than in normal pancreatic epithelial cells. The addition of LINC00261 reduced cell viability and invasion and facilitated apoptosis. miR-23a-3p was negatively correlated with LINC00261 levels, and high expression of miR-23a-3p indicated a low probability of survival. miR-23a-3p was the target of LINC00261 and weakened the effect of LINC00261 on the viability, invasion, and apoptosis of pancreatic cancer cells. In conclusion, LINC00261 over-expression suppressed cell viability and invasion and increased apoptosis by reducing the expression of miR-23a-3p in pancreatic cancer cells, indicating a new goal in the treatment of pancreatic cancer [92].

According to literature data, abnormal microRNA expression is associated with the development and progression of cancer. Zhu et al. study aimed to assess the functional role of miR1425p in migration and invasion and to investigate its molecular mechanism in pancreatic cancer cells [93]. In the first place, it was found that the expression of miR1425p is reduced in the tissues and cell lines of pancreatic cancer. In addition, the α catalytic subunit of phosphoinositide 3-kinase (PIK3CA) was identified as the target of miR1425p.

PIK3CA expression was elevated in tumor tissues and its expression was negatively regulated by miR1425p expression. miR1425p over-expression inhibited the proliferation, migration, and invasion of PanC1 cells, while PIK3CA reversed this inhibition. In addition, miR1425p inhibited the expression of the adhesion foci kinase (FAK) and matrix metaloproteinates 9 (MMP9), as well as the level of phosphorylated protein kinase B (AKT), while PIK3CA reversed the suppression induced by miR142-5p. In conclusion, miR1425p acts as a tumor suppressor, inhibiting the migration and invasion of pancreatic cancer by inhibiting the expression of FAK and MMP9, as well as the signaling pathway phosphathydylositol/AKT 3-kinase by targeting PIK3CA. These results suggest that miR1425p may be a new therapeutic target for the treatment of pancreatic cancer [93].

The relationship between the expression of the miRNA family and the cell cycle, including the disturbed cycle of the cancer cell, certainly requires further analysis. There is no doubt, however, that the molecules in question have great prognostic, predictive, and therapeutic potential. Promising research results have led scientists to look for new regulatory molecules. Long non-decoding RNA, or lncRNA, has been of particular interest recently.

Pancreatic cancer is one of the most common causes of cancer death in the world due to the absence of early symptoms, the occurrence of metastases and chemoresistance. Therefore, early diagnosis by detecting biomarkers, blocking metastases, and overcoming chemoresistance are effective strategies for improving the survival of patients with pancreatic cancer. A growing body of evidence indicates that long non-coding RNA (lncRNA) and circular RNA (circRNA) play an essential role in modulating the susceptibility to chemotherapy in pancreatic cancer [94]. lncRNAs play an important role in drug resistance in pancreatic cancer cells including HOTTIP, HOTAIR, PVT1, linc-ROR, GAS5, UCA1, DYNC2H1-4, MEG3, TUG1, HOST2, HCP5, SLC7A11-AS1, and CASC2. Moreover, also important is the function of a circRNA, such as circHIPK3 and circ_0000284, in regulating the sensitivity of pancreatic cancer cells to drugs. In addition, a number of compounds, including curcumin, genistein, resveratrol, quercetin, and salinomycin can modulate lncRNA expression and enhance chemosensitivity in pancreatic tumors. Therefore, aiming at specific lncRNAs and cicrRNAs may help to reverse the chemo resistance of pancreatic cancer cells [94].

“Liquid biopsy,” the recently adopted term for blood-based molecular analyses in the diagnosis of cancer patients, could provide innovative monitoring of disease evolution and response to treatment. Circulating microRNAs derived from cancer cells have been tested as potential substitutes or compliments for direct tissue biopsy. The main advantage of liquid biopsies is the ability to compare serial samples from the same patient and thus generate a real-time reading of molecular disease progression and response or resistance. Furthermore, blood collection is minimally invasive and provides biosamples of comparable composition from a homogeneous compartment, i.e., a blood stream. Circulating miRNAs provide separate and complementary information: altered patterns of miRNA expression in the circulation indicate a change in the steady state of the whole disease-carrying organism in response to treatment, as well as a response or progression of the disease. Cells export apoptotic bodies containing miRNAs, shedding vesicles and exosomes into the bloodstream [95,96,97,98].

Microbubbles are impermeable to RNases, which explains the remarkable stability of extracellular miRNAs. Additionally, most extracellular miRNAs in plasma or serum are devoid of membrane vesicles but bound to 1 of 4 proteins in the Argonaute family (AGO) [99]. The remarkable stability of the AGO2 protein explains the stability of bound miRNA even in environments rich in nucleases and proteases [98].

Data from multiple laboratories suggest that different RNA species can be specifically packaged into microbubbles using active sorting mechanisms that have not been fully elucidated [100]. Blood cells are the main factors contributing to the extracellular content of miRNAs in the circulation [101]. Different organs are involved in the circulation of miRNAs because tissue-specific miRNAs such as miRNA-122 (liver), miRNA-133a (muscle), miRNA-208a (heart), and miRNA-124 (brain) have been consistently detected in plasma samples [102,103,104]. Tumors release miRNAs into the bloodstream, and miRNAs specific to tumor tissue have been found in circulation at different stages of the disease.

Circulating miRNA trapped in microbubbles can be transferred to recipient cells as signaling molecules and alter gene expression in target cells [100]. In the circulation, the miRNA can function as hormones, and the miRNA in question can function as an oncogene or tumor suppressor gene depending on the cellular context and the target organ [105]. The concentration of miRNA in the bloodstream reflects changes in the homeostasis of the whole organism, and the miRNA panel can be easily quantified by PCR [106]. The results of such studies established the basis for circulating miRNAs as biomarkers of the disease. In recent years, there has been a wealth of information pointing to the potential use of circulating miR in pancreatic cancer screening [107]. In 2014, Ganepola et al. conducted an unbiased screening method to develop a panel of blood-based diagnostic biomarkers consisting of circulating miRNA for the detection of pancreatic cancer at an early stage. They compared 8 patients with early-stage pancreatic cancer with 11 healthy controls and performed high-performance screening using hybridization microarrays analysis [108]. miRNA-22, miRNA-642b and miRNA-885-5p were verified and evaluated as a diagnostic panel. They were found to give 91% sensitivity and 91% specificity. Marker CA19-9 showed 73% sensitivity and 100% specificity. Kojima et al. analyzed 571 serum samples taken from healthy individuals, patients with pancreatic, biliary, or other gastrointestinal cancers, and patients with benign abnormalities of the pancreas or biliary tract. It turned out that 8 miR (miR-125a-3p, miR-4294, miR-4476, miR-4530, miR-6075, miR-6799-5p, miR-6836-3p, and miR-6880-5p) achieved a sensitivity of 80.3%, a specificity of 97.6%, and an accuracy of 91.6% in the detection of pancreatic and biliary tract cancers compared to healthy controls, benign abnormalities, or other types of cancer [109].

There are reports beyond cancer-related miRNAs that show that similar miRNAs are indicators of benign pancreatic pathology. Chronic pancreatitis is a risk factor for pancreatic cancer and has the same clinical symptoms as pancreatic cancer. For example, circulating miR-155, 181a, 181b, 196a, 200a, 200b, and 212 were reported as significantly elevated in patients with chronic pancreatitis compared to healthy controls [75,80,110]. Li et al. showed that serum levels of miR-200a and miR-200b are similar in patients with pancreatic cancer and pancreatitis, although they are elevated compared to healthy controls [91]. Interestingly, the miR-200 family can inhibit the transition from the epithelium to mesenchymes and therefore may play a direct signaling role [98]. In conclusion, circulating miRNAs may indicate the presence of chronic inflammatory and potentially precancerous processes in the pancreas. Surgical removal of pancreatic cancer lowered circulating miR-221 in 8 patients [111], and miR-18a decreased significantly after surgical removal of pancreatic cancer in 9 patients [112]. It was noted that at the time of tumor recurrence in one patient, the levels of circulating miR-18a increased again despite the absence of any increase in the conventional tumor marker in the CA-19-9 serum. Additionally, circulating miR-483-3p levels decreased after surgery in 2 pancreatic cancer patients [113]. Li et al. [114] report that higher levels of miR-1290 predicted worse outcomes in patients undergoing pancreatoduodenectomy.

Drug therapy affects many organ systems. MiRNA concentrations in the circulation can serve as readily available markers of treatment effectiveness and even indicate pathways altered by a given treatment. Wang et al. showed that drug-induced liver damage can be indicated by a dramatic increase in plasma miR-122 and a decrease in miR-170. Circulating miRNA was found to be more sensitive markers of liver damage than alanine aminotransferase [115].

Shivapurkar et al. showed that circulating miR-296 is lost during tumor progression and correlates with metastases in colorectal cancer [116].

Patients with metastatic colon cancer were treated with a multidirectional receptor tyrosine kinase inhibitor, sunitinib, and the antimetabolite capecitabine. Circulating miRNAs were analyzed from 7 serum samples before and after treatment. Three patients had reduced miR-296 at 4 weeks after treatment. During this period, 4 patients had elevated levels of miR-296. Compared to the patients with longer survival and better clinical outcomes, patients with shorter survival and poor clinical outcomes showed a decrease in miR-296 levels after 4 weeks compared to baseline. The loss of miR-296 may be one of the mechanisms of primary resistance of colorectal cancer to chemotherapy, which can be translated into studies in pancreatic cancer patients using a similar treatment regimen.

One type of miRNA can regulate the expression of hundreds, and in some cases, it is believed to be as many as thousands. Moreover, one mRNA molecule may be regulated by different miRNAs. The complexity of the regulatory function of miRNAs is also argued by the fact that one miRNA can regulate different mRNAs in two ways: by degradation or translation repression. MiRNA may also regulate, depending on the type of tumor, oncogenes and suppressor genes [117], and may itself act as an oncogene or a suppressor.

In neoplastic diseases, the decreased expression level of miRNAs regulating the formation of oncogenes leads to their excessive production, while the increase of the level of other miRNAs, such as those involved in the regulation of the expression of suppressor genes, leads to inhibition of the formation of these anti-oncogenes [118].

The emergence of the latest technological developments and their application, such as next generation sequencing, individualized oligonucleotide-based microarray analysis, RT-PCR, in situ hybridization and Northern blotting using probes modified with blocked nucleic acid (LNA) provided a number of specific miRNA expression profiles that can be used for diagnosis [119,120,121,122,123,124,125,126,127,128].

The expression profiles of many miRNAs obtained from cancer tissues and the right evidence that they can be used in the prediction and diagnosis of cancer in patients in the world.

Differences in the expression of miRNAs in the peripheral blood have been noticed in patients with tumors such as: multiple myeloma, cancer of the nasopharynx, stomach, prostate, of the mammary gland, large intestine, pancreas, diffuse large B-cell lymphoma, squamous cell carcinoma, lung, ovarian cancer or neoplasm’s occurring within the central nervous system [127].

Observing such a risk can be helpful in diagnosing these cancers. All cancer cells have a significant ability to grow and share in the process of cancer transformation. In the process of neoplastic transformation, there are also changes in the systems controlling cell death, which may be e.g., under the influence of modulation of the gene expression profile.

MicroRNAs control the level of gene expression in order to be modulated, making it a promising object for research into novel targeted therapies.

It is claimed that miRNAs contribute to tumorigenesis by acting as oncogenes or suppressors and are capable of restoring normal gene expression profiles to suppress tumor growth [128].

Previous research results have been based on a related disorder of regulation in the expression of appropriate miRNAs with different types of cancer. The procedure is based on the identification of the miRNA profile in cancerous and non-cancerous tissues. The miRNA profile allows one to determine the degree of tumor development, which can be therapeutic and allows using the most appropriate therapy for the case.

An extremely promising thing to do in diagnosis is to discover that miRNA, which is a marker of the cancer process, does not require invasive diagnostic procedures. Intensive research is underway into the use of miRNAs present in the body (such as plasma, cerebrospinal fluid, saliva, urine, seminal fluid) as a diagnostic marker or as a prognostic marker of cancer.

It is also considered that miRNAs present in the blood stream are obtained from cancerous tissues as a result of the death of the cells and the release of miRNA steaks from their side. It is now known that it is also possible to secrete miRNAs externally in secretive bubbles or to combine them with proteins and lipoproteins.

It is now becoming apparent that miRNAs have the potential to be used as biomarkers to diagnose cancer. For example, some miRNAs such as miR-376a, miR-301, miR-155, miR-21, miR-221, and miR-222 are over-expressed in pancreatic cancer, and their expression is limited only to cancer cells without expression in normal cells [33]. In addition, it has been shown that the differentiated expression of miR-96 [129], miR-34a [130], and miR-21 [131] accurately distinguishes pancreatic cancer from normal adhering tissue.

Another study of the analysis of expression between normal pancreas and pancreatic cancer suggested that the presence of miR-216 and miR-217 and the absence of miR-133a are unique to a healthy pancreas [132].

Elevated expression of miR-103 and miR-107 in the presence of low miR-155 expression is a signature profile of pancreatic tumors [133]. A total of 20 miRNAs have been identified that can distinguish pancreatic cancer from chronic pancreatic and normal pancreatic diseases [134]. Analysis of miR-196a and miR-217 expression in a fine needle aspirate classified as malignant pancreatic cancer from benign lesions was performed [135]. Higher levels of miR-196a were observed in serum samples of pancreatic cancer compared to control [136]. Serum miR-196a may be a potential marker of pancreatic cancer and patient selection for laparotomy [106]. The 35 miRNAs identified in the PanIN-3 and miR-196b lesions proved to be the best biomarker for detecting these changes [137]. Pancreatic cancer patients with higher plasma levels of miR-221 showed a significant correlation with distant metastases [138]. An analysis of 735 circulating miRNAs in pancreatic cancer and control serum showed that miR-1290 had the best diagnostic performance among other upwardly regulated circulating miRNAs [111]. CA19-9 is now widely used as a standard marker of serum that identifies pancreatic cancer. However, its use is limited to monitoring response to therapy. CA19-9 is not a sensitive or specific diagnostic marker [114,139].

Interestingly, the combination of miR-16 and miR-196a with CA19-9 has been shown to be more accurate in distinguishing pancreatic cancer from normal tissue with sensitivity and specificity of 92.0% and 95.6% respectively.

Habbe et al. [140] identified miR-155 as a potential biomarker for detecting early stage pancreatic cancer. In conclusion, these studies highlight the potential of miRNA to be used as a valuable tool for distinguishing pancreatic tumors from normal pancreas and classifying the stage and degree of the tumor, alone or in combination with other biomarkers.

## 4. microRNAs in Prognostic Assessment of Pancreatic Carcinoma

As data are collected, it becomes increasingly clear that in addition to the importance of miRNA in the diagnosis of pancreatic cancer, it can also be used as potential prognostic biomarkers. Elevated levels of miR-21 have been shown to be associated with poor therapeutic outcomes in patients undergoing gemcitabine therapy [141]. In addition, it has been reported that miR-21 over-expression in pancreatic ductal adenocarcinoma (PDAC) is correlated with shorter overall survival in patients with negative lymph nodes and is strongly associated with liver metastases [142]. Interestingly, it has been observed that patients with low miR-21 expression benefit from gemcitabine treatment [132].

There are studies that highlight the importance of miRNAs in predicting pancreatic cancer. Reduced expression of miR-204 and miR-142-5p was demonstrated in samples of a gemcitabine-resistant pancreatic tumor. Studies have found a positive correlation between miRNA and prolonged survival of pancreatic cancer patients. miR-142-5p has been classified as a predictive marker of gemcitabine response [143]. Studies have shown that a pool of six miRNAs can distinguish survivors who have lymph node metastases, dying within 2 years. The above study suggested that high levels of miR-196a-2 could predict poor survival [144].

MiR-155, miR-200, miR-203, miR-205 [133], miR-212 and miR-675 [137] miR-200c [145], miR-21 [136] and reduced expression of miR-34a, miR-30d [136], miR-130b [146], miR-148a, miR-187, and let-7g [144] in pancreatic cancer are known to be associated with worse survival rates. miR-203 has been identified as a new prognostic marker for patients with pancreatic adenocarcinoma who have not undergone resection [147]. Expression of miR-155, miR-196a, and miR-10b was correlated with increased invasiveness and poor overall survival of pancreatic cancer patients [148]. In addition, a poor prognosis of pancreatic cancer was also observed in patients expressing high levels of miR-17-5p clusters.

## 5. microRNA–Therapeutic Potential

miRNAs are potential targets for therapeutic interventions [149]. miRNA-based anticancer therapies mainly involve the introduction or restoration of tumor suppressors as “miRNA followers” and target oncogenic miRNAs using miRNA antagonists. miRNA copycat is a chemically modified double stranded miRNA that is used to miRNA function, which is regulated down or reduced due to pancreatic cancer [150].

Kent et al. demonstrated that transduction via the tumor suppressor miRNA virus, miR-143/145, inhibited tumor growth in pancreatic cancer cells [151]. The supply of miR-143 via adenovirus also showed inhibitory effects in pancreatic cancer cells by blocking metastases [152].

Śrivastava et al. report that the restoration of miR-150 can significantly inhibit malignant potency and growth of pancreatic cancer cells [153]. Similarly, the addition of miR-34 showed promising results, not only inhibiting the growth of pancreatic cancer cells, but also increasing their sensitivity to chemo- and radiotherapy [81].

A separate approach involves miRNA antagonists. These are single-pot antisense oligonucleotides corresponding to the target miRNA, chemically synthesized with a specific modification to ensure high stability, binding affinity, and protection against nucleases [131,154].

They are complementary to the leading strand to the target miRNA and inhibit its activity by binding to the seed region or by interfering with miRNA biogenesis [155,156].

Along with these antagonistic miRNAs, small molecule inhibitors are also positively used for aiming at miRNAs in vitro [157,158].

Aiming at oncogenic miR-21 with a specific fine molecule antagonist yielded promising results and showed inhibition of cell growth and proliferation in PDAC cells [159].

Similarly, the silencing of miR-10a effectively inhibits metastases in pancreatic cancer cells and primary human tumors [160].

The repression of miR-212 and miR-132 using antagonistic miRNAs also inhibited cancer growth [161].

Passadouro et al. demonstrated that the joint delivery of human albumin-1-palmitoilo-2-oleoilo-sn-glycerol-3-ethylphosphocholine: cholesterol with anti-miR oligonucleotide effectively suppressed the upregulation of miRNA pancreatic cancer (miR -10, miR-21, miR-221, and miR-222) [162,163].

As a cytotoxic drug discovered more than 60 years ago, 5-fluorouracil (5-FU) is still widely used in the treatment of neoadjuvant, adjuvant, or metastatic various cancers. Although 5-FU is the first approved chemotherapeutic drug and clinical treatment for first-line pancreatic cancer, it can only slightly prolong the survival of patients. Many carcinogenic or suppressive miRNAs have been found to be associated with resistance to 5-FU in pancreatic cancer [164].

Many oncogenic miRNAs, such as miR-21, miR-221, and miR-320a, may promote resistance to 5-FU in pancreatic cancer cells. miR-21 can aim at PTEN and PDCD4 via the PI3K/AKT/mTOR route. miRNA-21 promotes tumor proliferation and increases resistance to 5-FU in human PDAC. Moreover, upward regulation of PTEN and PDCD4 may weaken the effect of miR-21 on pancreatic cancer resistance on 5-FU [165].

MiR-320a expression has been shown to be significantly increased in pancreatic cancer cells resistant to 5-FU. miR-320a stimulates pancreatic cancer cells to reveal the mesenchymal phenotype. In addition, it increases their ability to attack cells and migrate.

miR-320a may stimulate resistance to 5-FU by binding to 3′UTR mRNA PDCD4 in pancreatic cancer [166]. miR296-5p promotes the invasion and migration of EMT-related cells. miR296-5p induces resistance to 5-FU by inhibiting BOK expression in pancreatic cancer cells [167]. MiR-146a-5p was found to be significantly elevated in pancreatic cancer cells. It can regulate carcinogenesis and chemoresistance of pancreatic cancer by inhibiting the expression of TRAF6 in vivo, which is also a potential biomarker of 5-FU chemotherapy [168]. In the case of miR-183, it can inhibit the growth of pancreatic cancer cells and sensitize cells to 5-FU by targeting the PTEN/PI3K/Akt pathway [169]. MiR-34a can make pancreatic cancer cells much more sensitive to chemotherapy and radiation therapy. In addition, it can inhibit cancer growth and induce cell apoptosis by inhibiting the expression of Notch1, Notch2, Notch4, and Bcl-2. The use of miR-34a in in vitro studies by injecting it sensitizes the antitumor effects of 5-FU [170]. Cisplatin (DDP) drug used in combination chemotherapy has features of a strong antitumor effect. There are already several studies investigating the relationship between miRNA and cisplatin resistance in pancreatic cancer.

Inhibition of miR-1180 may potentiate cisplatin-induced pancreatic cell apoptosis, causing cisplatin resistance [171]. In turn, high miR-100 expression increases cisplatin sensitivity by lowering the level of MRNA FGFR3 expression in pancreatic cancer [172].

Download regulation of miR-374b is another important factor for acquired resistance to cisplatin in pancreatic cancer, and the high ectopic expression of miR-374b in cisplatin-resistant pancreatic cancer cell lines may reduce drug resistance [173]. By aiming at the expression of Notch, C-Met, and Bcl-2, miR-34a can increase the ability of cancer cells to self-renewal and survival. It has been found that the expression of miR-34a, which can regulate apoptosis via p53, is noticeably reduced in pancreatic cancer.

Doxorubicin (Dox), also known as hydroxydaunorubicin, Adriamycin, and hydroxydaunomycin, is one of the most effective broad-spectrum cancer drugs and is commonly used in cancer chemotherapy [174]. Literature data show that some miRNAs (miR-142, miR-137, miR-9, and miR-212) are also associated with resistance to Dox in pancreatic cancer [175,176,177].

Apart from GEM, 5-FU, cisplatin, and Dox, several miRNAs are also involved in resistance to some other drugs for treating pancreatic cancer such as: oxaliplatin, paclitaxel, erlotinib, lapatinib [178]. To develop microRNA-based therapeutics that may improve the prognosis of pancreatic carcinoma subjects, additional investigations using translational research and clinical trials are urgently needed.

## 6. Summary

One type of miRNA can regulate the expression of hundreds, and it is assumed that in some cases even thousands of genes. In addition, one mRNA molecule can be regulated by different miRNAs. The complexity of the regulatory function of miRNAs is also supported by the fact that one miRNA can regulate different mRNAs in two ways: by their degradation or by repression of translation. MiRNA can also regulate, depending on the type of cancer, oncogenes, and suppressor genes [179], and it can also behave like an oncogene or suppressor. In cancer, a reduced level of expression of miRNAs regulating the formation of oncogenes leads to their excessive production, while an increase in the level of other miRNAs, those that are involved in the regulation of the expression of suppressor genes, leads to the inhibition of the formation of these anti-oncogenes [118]. Expression profiles of multiple miRNAs obtained from cancerous and normal tissues prove that they can be used in the prognosis and diagnosis of cancer in patients. By examining miRNA profiles, the presence of a constant concentration of miRNA in human peripheral blood was demonstrated. Differences in miRNA expression in peripheral blood have been noted in patients with cancers such as multiple myeloma, nasal throat, stomach, prostate, mammary gland, large B-cell lymphoma, squamous cell carcinoma, lung cancer, or cancers occurring within the central nervous system [119]. Observation of such differences may be helpful in the diagnosis of these cancers.

All cancer cells are characterized by increased ability to grow and divide. In the process of cancer transformation, there are also changes in the systems controlling cell death, which can happen, for example, under the influence of modulation of the gene expression profile.

MicroRNAs control gene expression levels, making them a promising object of research in novel targeted therapies. It is postulated that miRNAs contribute to the formation of tumors by acting as oncogenes or suppressors and are capable of restoring the normal gene expression profile, in order to stop the development of the tumor [33,128]. Previous research results indicate a link between regulatory disorders in the expression of relevant miRNAs and the occurrence of various types of cancer. Owing to the development of research on microRNAs, the possibility of typing cancers has appeared. The procedure is based on the identification of the miRNA profile in tumor tissues and unchanged disease. The differentiation and expression profile of miRNA allows to determine the degree of tumor development, which clarifies the therapeutic possibilities and would allow the use of the most appropriate therapy for a given case. Extremely promising in diagnostics is the discovery that miRNAs, which are markers of the cancer process, do not require invasive diagnostic procedures. Intensive research is underway on the use of miRNA present in body fluids (such as plasma, cerebrospinal fluid, saliva, urine, seminal fluid) as a diagnostic marker or prognostic marker of cancer. Initially, it was believed that miRNAs present in the bloodstream get there from cancer tissues as a result of cell death and the release of miRNA molecules from inside them. It is now known that it is also possible to secrete miRNAs outside the cell in secretary vesicles or combine them with proteins or lipoproteins.

## Figures and Tables

**Table 1:** The role of miRNA in the metabolism of cancer-transforming cells [28,29].

Function	microRNA
Inhibition of proliferation	miR17-92, miR-21
Energy metabolism disorders	let-7, miR-15b, miR-21, miR-23a/b, miR-155
Inhibition of anti-oncogene action	miR-21, miR-126
Avoiding elimination by immune cells	miR-21, miR-155, klaster miR17-92
Replication immortality	let-7, miR-10b, miR-16, miR-21, miR-221/222
Promoting inflammation	let-7d, miR-21, miR-23b, miR-126, miR-155, miR-200c
Activate metastasis	let-7d, miR-10b, miR-15b, miR-21, miR-29
Angiogenesis induction	let-7, miR-15b, miR-21, miR-125, miR155, miR-200, miR17/20/106
Accumulation of mutations, instability of genetic material	miR-21, miR-15b, miR-155

**Table 2:** Changes in miRNA expression profile for various cancers.

Type of Cancer	microRNA	References
lung cancer	miR-21, miR-191, miR205, miR-210, miR-214	[34,35,36]
breast cancer	miR-125b, miR-145, miR-21, miR-155	[37]
pancreatic cancer	miR-155, miR-21, miR-221, miR-222, miR-376a, miR-301	[38,39,40,41,42,43]
ovarian cancer	miR-200a, miR-141, miR-200c, miR-200b, miR-199a, miR-140, miR-145	[44,45,46,47]
prostate cancer	miR-125b, miR-145, miR-224, miR-23b, miR-222	[48,49]
skin cancer	miR-203, miR-205, miR-200c	[50]
colorectal cancer	miR-181a/b, miR-135a/b, miR-150, miR-150-5p, miR-155, miR-181b, miR-200 a/c, miR-22, miR-106a, hsa-miR-103a, hsa-miR-1827, miR-135b, miR-150, miR-150-5p, miR-181b, and let-7f-5p, miR-323a-3p, miR-382-5p, and miR-376a-3p miR-30c-5p/TCF7	[51,52,53,54]
liver cancer	miR-145, miR-198, miR-222, miR-224	[55,56,57,58]
kidney cancer	miRNA-203, miRNA-32, miRNA-15a, miR-17-5p–miR-25-3p	[59,60]
thyroid cancer	miR-221, miR-222, miR-146b, miR-15a, miR-155	[61,62,63,64]

**Table 3:** Changes in the expression of circulating microRNAs in pancreatic cancer [72].

Circulating miRNAs	Expression Profile
miR-21, miR-155, miR-196a, miR-210, miR-16, miR-21, miR-155, miR181a, miR-181b, miR-196a, miR-210, miR-26b, miR-34a, miR-122, miR-126, miR-145, miR-150, miR-196a, miR-223, miR-505, miR-636, miR-885.5p, miR-18a, miR-21, miR-221, miR-483-3p, miR-20a, miR-21, miR-24, miR-25, miR-99a, miR-185, miR-191, miR-1246, miR-4644, miR-3976, miR-4306	Up-regulated
let-7d	Down-regulated

## Data Availability

Data sharing is not applicable to this article.
